# Reconstruction algorithms for grain mapping by laboratory X-ray diffraction contrast tomography

**DOI:** 10.1107/S1600576722010214

**Published:** 2022-12-01

**Authors:** Haixing Fang, Wolfgang Ludwig, Pierre Lhuissier

**Affiliations:** a Université Grenoble Alpes, Grenoble INP, CNRS SIMaP, 38402 Grenoble, France; b European Synchrotron Radiation Facility (ESRF), 71 Avenue des Martyrs, 380000 Grenoble, France; c Université de Lyon, INSA Lyon, CNRS MATEIS, 69621 Villeurbanne, France; Argonne National Laboratory, USA

**Keywords:** diffraction contrast tomography, grain mapping, reconstruction algorithms, 3D imaging, forward calculations, back calculations

## Abstract

X-ray-based non-destructive 3D grain mapping techniques are well established at synchrotron facilities. To facilitate everyday access to grain mapping instruments, laboratory diffraction contrast tomography (LabDCT), using a laboratory-based conical polychromatic X-ray beam, has been developed and commercialized. Yet the currently available LabDCT grain reconstruction methods are either ill-suited for handling a large number of grains or require a commercial licence bound to a specific instrument. To promote the availability of LabDCT, grain reconstruction methods have been developed with multiple reconstruction algorithms based on both forward and back calculations. The different algorithms are presented in detail and their efficient implementation using parallel computing is described. The performance of different reconstruction methods is assessed on synthetic data. The code to implement all the described algorithms has been made publicly accessible with the intention of fostering the development of grain mapping techniques on widely available laboratory instruments.

## Abstract 2

Grain reconstruction methods based on both forward and back calculations have been developed for laboratory-based diffraction contrast tomography. These methods are computationally efficient and can give good orientation and spatial accuracies, and the code is open source.

## 1. Introduction

The production of high-performance metals and alloys has benefitted substantially from a better understanding of the fundamental processes, such as phase transformation and recrystallization (Juul Jensen & Zhang, 2020 ▸). Microstructure at the level of grains, grain boundaries and interfaces is often tuned or even engineered to optimize material properties. To facilitate this understanding and control of the microstructure, 3D characterization of grain orientations, volumes, shapes and grain boundary characteristics, termed grain mapping, is becoming increasingly popular (Miller *et al.*, 2020 ▸). Based on diffraction imaging, a number of X-ray grain mapping techniques have been developed at synchrotron facilities for non-destructive characterization of grain structures in 3D. These include three-dimensional X-ray diffraction (3DXRD; Poulsen, 2004 ▸; Suter *et al.*, 2006 ▸; Hayashi *et al.*, 2019 ▸), diffraction contrast tomography (DCT; Ludwig *et al.*, 2008 ▸, 2009 ▸), differential aperture X-ray microscopy (Larson *et al.*, 2002 ▸) and X-ray dark-field microscopy (Simons *et al.*, 2015 ▸), offering immense possibilities for 3D grain mapping and sometimes even strain mapping over a broad range of length and temporal scales (Poulsen, 2020 ▸). However, all these techniques require the use of synchrotron radiation, placing a serious limitation in terms of access to these instruments.

To broaden the use of grain mapping techniques and overcome such limited access, laboratory-based X-ray diffraction contrast tomography (LabDCT), adapted from synchrotron DCT, has been developed (King *et al.*, 2013 ▸, 2014 ▸; van Aarle, Ludwig *et al.*, 2015 ▸) and commercialized (McDonald *et al.*, 2015 ▸, 2017 ▸). Unlike synchrotron DCT using a monochromatic parallel beam, LabDCT uses a polychromatic conical beam (with its size usually confined by an aperture) to illuminate the sample. Transmitted diffracted beams fulfilling Bragg’s diffraction condition are recorded by the outer area of a 2D detector and give rise to diffraction spots corresponding to different reflections of the illuminated grains, whereas the direct transmitted beam is blocked by a beamstop placed in the central part of the detector. A grain map is then reconstructed from a series of diffraction images recorded for a stepwise rotation of the sample over 360° around a vertical axis.

While it does not require complex modifications of hardware on a conventional tomography setup other than positioning the aperture and beamstop, the grain reconstruction algorithm is rather complex for LabDCT grain mapping due to the use of a conical white beam. A first complication is that the photon energies for each diffraction spot are *a priori* unknown, and secondly, the crude approximation that all diffraction events occur at the sample centre (a common assumption for far-field diffraction setups) becomes invalid. Nevertheless, several grain reconstruction approaches have been reported. The very first reconstruction approach developed for a magnified geometry (sample-to-detector distance longer than sample-to-source distance, *L*_sd_ > *L*_ss_) uses Friedel-pair spots for indexing and then adopts an iterative algebraic reconstruction for grain shapes (King *et al.*, 2013 ▸). However, it can only deal with a moderate number of grains in the illuminated sample volume because of the need for explicit identification of the Friedel pairs over rotation angles, *i.e.* spot overlapping should be minimal. The later commercialized grain reconstruction approach [*GrainMapper3D* software (XnovoTech), initially working in a Laue focusing geometry, *L*_sd_ = *L*_ss_] is based on forward modelling and has been shown to perform rather well in reconstructing grain orientations and shapes for various types of sample using multiple scanning strategies (Bachmann *et al.*, 2019 ▸; Odders­hede *et al.*, 2022 ▸). However, detailed implementation of this approach has not been reported, and it has been restricted to a specific instrument and requires a commercial licence, preventing its use on other, more widely available, laboratory instruments.

To give a true boost to the use of grain mapping by LabDCT, we have developed grain reconstruction methods based on forward and back calculations. These methods share the characteristic of working on binarized images, where spot intensities are neglected, and reconstructing the grains one by one. In this paper, we present algorithms for indexing and growth (assigning the indexed orientation to the surrounding voxels) and show how to implement efficient computation using modern parallel GPU computing. As backbones for the reconstruction algorithm, the principles of the forward and back calculations for a general geometric configuration are described in detail in Appendix *A* [App appa]. Lastly, we validate and compare performances of various grain reconstruction methods using a synthetic grain structure and the corresponding simulated LabDCT projections generated by a forward projection model in both Laue focusing and magnified geometries. In a follow-up paper, we will demonstrate the implementation of these grain reconstruction methods for real experimental samples measured on a conventional tomography setup and evaluate the accuracy on the basis of comparisons with synchrotron DCT measurements. To allow scientists to implement grain mapping on their own tomography setups (primarily for X-rays but can also be extended to neutrons), we have made the code for grain mapping by LabDCT publicly accessible.

## 2. Algorithms for grain reconstruction

### 2.1. Overall procedure

**Figure 1:**
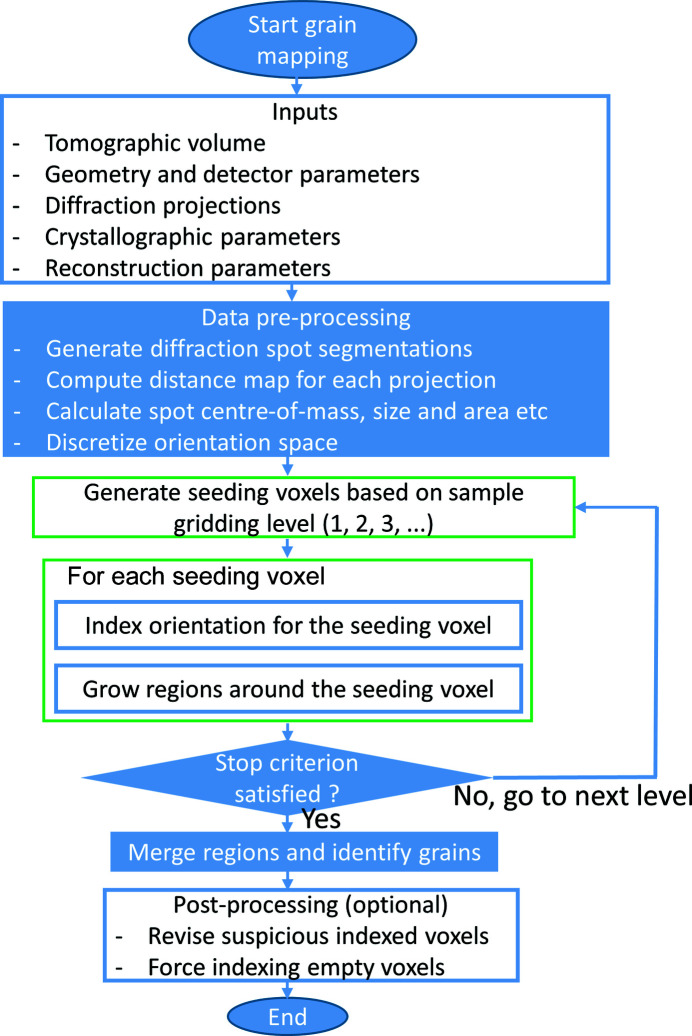
A flowchart for LabDCT grain reconstruction.

Fig. 1 ▸ shows a flowchart of the overall procedure for the grain reconstruction method developed in this work. Note in particular that LabDCT can so far only deal with grains with negligible lattice strain and small intragranular orientation spread, although a recent study theoretically attempted to estimate elastic strain from synthetic LabDCT data (Lindkvist & Zhang, 2022 ▸). In this work, we limit our grain reconstruction method to strain-free samples with known crystallographic lattice parameters. To start a grain mapping, the following inputs are required:

(i) A sample volume obtained from absorption tomographic reconstruction, which is segmented to provide a volume mask for determining where to reconstruct grains.

(ii) Geometric information, including *L*_ss_, *L*_sd_, det*y*0, det*z*0, *S_y_*, *S_z_*, φ_*x*_, φ_*y*_ and φ_*z*_ (see notation definitions in Appendix *A* [App appa]), and detector parameters including pixel pitch, width and height. It is not necessary for the geometric information to be accurate in the first run as it can be refined by fittings after a grain reconstruction result is obtained.

(iii) Diffraction projections at each rotation angle.

(iv) Lattice parameters of the sample.

(v) Self-defined reconstruction parameters, including the number of {*hkl*} families for indexing so a list of (*hkl*) can be computed, minimum completeness (*C*_min_), trust completeness (*C*_trust_), maximum acceptable median distance between forward projected and experimental spots (max*D*_median_), maximum acceptable distance of completeness weighted centres (max*D*_centre_), and drop-off parameter (δ_drop-off_), which controls the growth of the region around an indexed seeding voxel. Here, the completeness is defined in the same way as in many other articles (*e.g.* Bachmann *et al.*, 2019 ▸), *i.e.* the number of forward signals intersecting with experimental signals divided by the number of theoretical expected signals. *C*_min_ and max*D*_median_ are used to control whether an orientation indexing can be accepted or not; max*D*_centre_ is the distance tolerance (typically set as ∼3 pixels) below which updating of the centre of the grown region is stopped. More details of the reconstruction parameters are presented in Section 2.2.

The LabDCT projections are then preprocessed, *e.g.* by a rolling median to remove most of the background noise (Ludwig *et al.*, 2008 ▸), and binarized to segment the diffraction spots as accurately as possible. From these binarized images, distance maps are computed (nearest Euclidean distance to find a spot signal for deriving *D*_median_ during subsequent indexing) and spot features such as centre of mass and size are calculated. The orientation space of a fundamental zone for a specific type of crystal symmetry is discretized to have a maximum neighbouring misorientation of 2 or 1°, depending on the subsequent indexing method. In this work, we adopt the hyperspherical orientation sampling method developed by Larsen & Schmidt (2017 ▸) as it provides a reduced number of orientations and generates a better overall distribution of misorientation errors compared with the random or nearly uniform sampling methods (Quey *et al.*, 2018 ▸; *MTEX* toolbox, Bachmann *et al.*, 2011 ▸).

Seeding voxels are generated by controlling the minimum allowed distance between them. This minimum allowed distance is first assigned a relatively large value and then decreases with iterative reconstruction procedures, which corresponds to an increase in the number of seeding voxels. The degree of fineness of the seeding voxel is referred to as the sample gridding level, starting from level 1 (coarse) to typically 10 or above (fine). During each reconstruction iteration, the orientation for each seeding voxel is indexed and the same orientation will be assigned to neighbouring voxels if the computed completeness and *D*_median_ are met with the preset criteria. The latter operation is termed region growth. When all the seeding voxels have been tested for one sample gridding level, a stop criterion, usually defined as the minimum acceptable indexed volume fraction, is checked to determine whether to continue generating new seeding voxels with a finer gridding level or to exit for subsequent merging of regions. Individual grains are identified from these merged regions with misorientation smaller than a preset value (typically 0.5°). After that, an optional procedure is to revise ‘suspicious’ indexed voxels (*e.g.* those forming very small grains) or force indexing for empty voxels, both achieved by comparing completeness values using indexed orientations from nearby voxels. In the following, we present algorithms for the indexing and growth in detail.

### 2.2. Indexing and growth

Given a seeding voxel *i*, the indexing aims to find an orientation that gives maximum completeness (*C*_max_) for this voxel. During the subsequent growth step, this orientation is carried over to neighbouring voxels, provided that (i) the completeness stays above a certain percentage of *C*_max_ and (ii) *D*_median_ of the voxel is not larger than the value previously assigned to it. Initially, the completeness values for all voxels are assigned to 0 and a relatively large value (*e.g.* 20 pixels) is assigned to *D*_median_.

**Figure 2:**
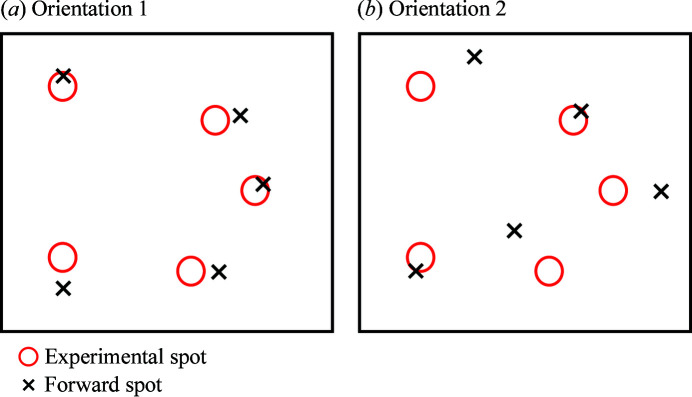
A schematic diagram illustrating the guidance of *D*_median_ for choosing the orientation between two candidates for accepting a voxel into a grown region. Both orientations give the same completeness (*C* = 2/5 = 0.4), whereas *D*_median_ is smaller for orientation 1 than for orientation 2. Therefore, orientation 1 is assigned to this voxel as long as the completeness value fulfils the growth criterion.

The indexing comprises three steps: (i) find promising candidates from all the orientations sampled from the whole orientation space; (ii) sample orientations in a local orientation space around each candidate (resulting in typically *N*_local_OR_ = 126 orientations with a misorientation of up to 2.5°) and forward compute completeness values, from which we choose the one giving *C*_max_ as starting value for a subsequent fit; and (iii) fit the orientation in order to maximize the completeness further to find the solution, *i.e.* *U_i_* = argmax*C*(seed *i*; *U*), where *U* expresses the orientation. During the subsequent growth step, the spot median distance, *D*_median_(seed *i*; *U_i_*), is calculated between all the forward spots expected on the detector and each nearest experimental spot, similar to the approach used by Raventós *et al.* (2019 ▸). When the completeness is high (*C* ≥ 0.5), *D*_median_ tends to be small or equal to 0 and this parameter does not play a role in either indexing or growth. Conversely, when the completeness is small (*C* < 0.5, *e.g.* close to a grain boundary), *D*_median_ is typically larger than zero and exhibits gradients which continue to provide guidance for the growth process. Fig. 2 ▸ shows a schematic diagram to illustrate the effect of *D*_median_ on choosing orientations when they give the same completeness values.

For the primary step of the indexing, two approaches have been developed, based on forward and back calculations, respectively. Using the forward calculation, orientations are ranked according to the completeness values and candidates are selected from the top ones (typically *N*_candidates_ = 50). Using the back calculation, orientations are ranked by the number of matched diffraction vectors (they are matched if the angle between 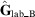 calculated for the spot centre of mass and 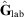 is smaller than a certain angle, typically set as 1°) and candidates are selected from the top ones.

If *C*(seed *i*; *U_i_*) < *C*_min_ or *D*_median_(seed *i*; *U_i_*) > max*D*_median_, the indexing is rejected and a new seed voxel is chosen. Otherwise, the indexing is accepted and growth starts using an algorithm similar to that of Bachmann *et al.* (2019 ▸). Complete­ness values for neighbouring voxels, *C*(voxel *j*; *U_i_*) and *D*_median_(voxel *j*; *U_i_*), are computed. When *C*(voxel *j*; *U_i_*) > *C*(seed *i*; *U_i_*) × (1 − δ_drop-off_) and *D*_median_(voxel *j*; *U_i_*) is not greater than the existing median distance value for voxel *j*, *U_i_* is assigned to voxel *j* to grow the indexed region. This process continues until no further voxel neighbouring the indexed region fulfils the growth criterion. After that, a completeness weighted centre-of-mass position is calculated and the distance between the centre and the seeding position is compared with max*D*_centre_. If it is smaller, the indexing and growth stage for this seeding voxel is finished; otherwise, the seeding voxel is replaced by the new centre and then re-indexed. For the re-indexing, calculations on all the orientations [as shown above in step (i) of the indexing] are not required and only calculations on orientations gridded over the local orientation space around *U_i_* are performed, because *U_i_* is already very close to the optimal solution. This process continues until the distance between the new centre and the previous centre is smaller than max*D*_centre_, resulting in a final centre position expected to be close to the grain centroid.

### 2.3. Implementation of indexing and growth

**Figure 3:**
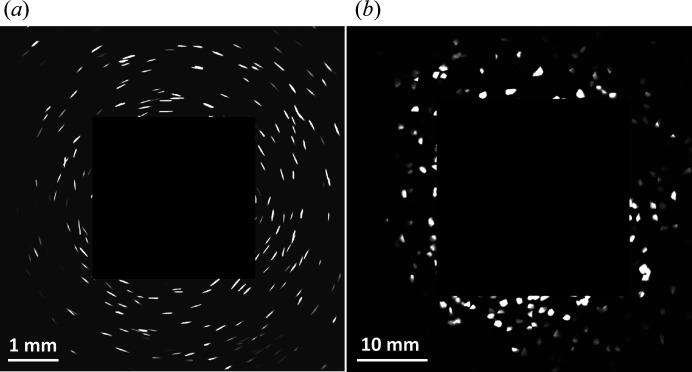
Forward projections simulated under (*a*) Laue focusing geometry and (*b*) magnified geometry using a virtual input grain structure, shown in Fig. 6. Diffraction spots have line shapes due to the Laue focusing effect in panel (*a*) and they resemble grain shapes better in panel (*b*).

The essential point of the indexing is to rank the orientation candidates that are close to the true solution as high as possible. Thus, the number of candidates can be chosen to be minimal for subsequent orientation refining, thereby significantly reducing the computational cost. To test the ranking, we generated an artificial Fe sample containing 144 grains with an average equivalent spherical diameter of 98.7 µm and a random distribution of orientations. This virtual sample is the same as that reported by Fang *et al.* (2020 ▸). Using a forward simulation model (Fang *et al.*, 2020 ▸), 181 LabDCT projections were simulated under a Laue focusing geometry and 121 projections for a magnified geometry (see Table 1 ▸ for geometry information). For both simulations, the diffraction spots for the first four {*hkl*} families were computed and an anisotropic point spread function was used to distribute the intensity from each diffraction event onto the detector pixels. An intensity threshold was applied to the diffraction images to remove spots with low intensities, mimicking the reality that signals that are too weak cannot be detected. These two types of simulated data correspond to typical experimental data sets acquired on a Zeiss Xradia 520 Versa commercial setup and on a conventional tomography instrument in the SIMaP laboratory (Fang *et al.*, 2022 ▸), respectively. Fig. 3 ▸ shows the LabDCT projections simulated under the two geometries.

**Figure 4:**
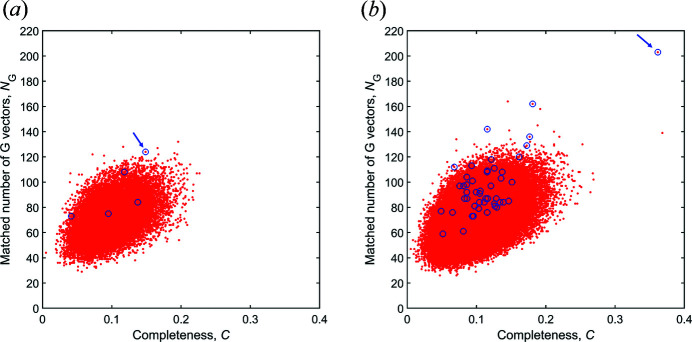
Computed completeness (*C*) and matched number of 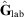 (*N*_*G*_) using all the discretized orientations for grain No. 1 in the virtual Fe sample in magnified geometry. (*a*) 32 768 orientations with an average misorientation of 2°. (*b*) 262 144 orientations with an average misorientation of 1°. Blue circles highlight the orientations close to the true grain orientation (misorientation < 3°). The arrow in panel (*a*) indicates that the candidate ranks fourth according to *N*_*G*_ and 1359th according to *C*, while that in panel (*b*) indicates that the candidate ranks first according to *N*_*G*_ and second according to *C*. With subsequent computations for further discretized orientations over the local orientation space and fitting, the completeness ultimately increases to 0.78.

Given the simulated data as ground truth, we randomly select one grain to test the indexing. Fig. 4 ▸ shows values of completeness (*C*) and matched number of 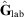 (*N*_*G*_) for all the orientations discretizing the whole fundamental zone with a misorientation of 2 and 1°. It can be seen in Fig. 4 ▸(*a*) that a potential orientation candidate can be ranked at the top according to *N*_G_ for 2°, whereas this orientation is ranked relatively low according to *C*. However, the potential candidate can be ranked at the top by *C* if a finer 1° discretization is used [Fig. 4 ▸(*b*)]. This indicates that ranking by *C* requires a much higher accuracy of the discretized orientation space, *i.e.* a higher number of orientations, than ranking by *N*_*G*_ to find a promising candidate close to the true orientation. In general, we find that the potential candidates can be ranked in the top 50 by *N*_*G*_ for both 2 and 1° discretization, while ranking by *C* requires 1° discretization to make the potential candidates lie in the top 50.

Following the selection of potential orientation candidates on the ranking list, finer discretization over the local orientation space and subsequent fitting for maximizing *C* are performed, as described in Section 2.2. The reason for further discretization is that good and robust fitting results can only be achieved when the starting input of the orientation is within about 0.5° of the true orientation. In this way, successful indexing can usually be achieved except for voxels located close to grain boundaries.

After successful indexing, neighbouring voxels can be assigned to the indexed seeding orientation by comparing *C* with the seeding voxel and *D*_median_ with their previous values. Unlike previous reports considering *C* only, we have introduced *D*_median_ to improve the accuracy of the growth, especially for voxels near the grain boundary. This is because competing for a smaller *D*_median_ helps to suppress false orientation assignments caused by scenarios where the completeness values still meet the growth requirement because forward calculated spots happen to coincide with experimental spots, especially for the case with crowded projection images (see the sketch in Fig. 2 ▸). Using the same data as shown in Fig. 3 ▸, we computed *C* and *D*_median_ for all voxels within grain No. 1 using its true orientation, as well as using any one of the orientations of the neighbouring grains. The results show that about 4% of voxels have a higher *C* with wrong neighbouring orientations than with their own correct orientation, whereas the proportion can be reduced to about 2% when computing both *C* and *D*_median_. This justifies the choice of including *D*_median_ in the computation. Note that this estimate is based on a random neighbouring misorientation case, representing an average level of inaccuracies.

### 2.4. Parallel computing for efficient computation

There are three main procedures limiting the computational speed for each run of indexing and growth: (i) ranking to search for potential orientation candidates; (ii) computing locally sampled orientations and fitting the orientation; and (iii) growing the indexed region by assigning the seeding orientation to neighbouring voxels that fulfil the growth criteria. For the first step by *N*_*G*_ ranking with back calculation, the computation cost scales with *N*_OR_ × *N*_proj_ × *N*_spots_ × *N*_*hkl*_, where *N*_OR_, *N*_proj_, *N*_spots_ and *N*_*hkl*_ are the numbers of discretized orientations, projections, spots per projection and *hkl* reflections, respectively, while the computation scales with *N*_OR_ × *N*_proj_ × *N*_*hkl*_ by *C* ranking with forward calculation. For *C* ranking the computation does not depend on *N*_spots_ but it requires a much higher *N*_OR_ compared with *N*_*G*_ ranking.

For a typical LabDCT data set as shown in Fig. 3 ▸ with the magnified geometry, *N*_proj_ = 121, *N*_spots_ ≃ 210 and *N*_*hkl*_ = 40, and vectorized computation of both *N*_*G*_ and *C* rankings for one orientation takes about 0.05 s using one single CPU core (Intel i7-10700). This means it would take 1638 s to compute 32 768 orientations and 13 107 s for 262 144 orientations using one CPU core. To reduce such long computation times to a more realistic number, parallel computing is thus required. Using an eight-core CPU such as is readily available nowadays, the computation time for *N*_*G*_ ranking can be reduced to about 200 s. To accelerate the computation further, we used GPU computing (NVIDIA Tesla V100-PCIE 32 GB) with CUDA programming (no further tuning for specific hardware) and reduced the time to about 2 s for both *N*_*G*_ and *C* rankings. For samples with lower symmetry than cubic, the computation time will increase but remain in the range of seconds using GPU computing. For subsequent computations for locally sampled orientations and fitting, the computation scales with *N*_candidates_ × *N*_local_OR_, where typically *N*_candidates_ = 50 and *N*_local_OR_ = 126. The computing time here can also be reduced to about 2 s using a GPU.

**Figure 5:**
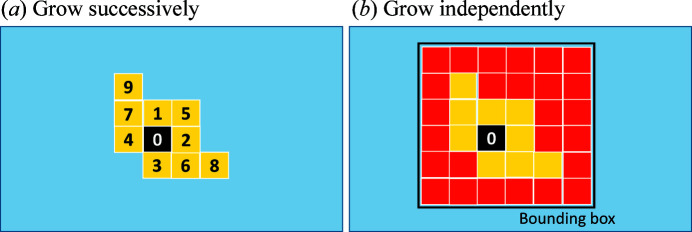
Sketches showing region growth around the indexed seeding voxel (coloured black and numbered 0). (*a*) The voxels grow successively, with one neighbouring voxel checked each time. The numbers represent a probable order of growth. (*b*) All the voxels within a bounding box are tested. The ones fulfilling the criterion are allowed to grow (marked yellow), whereas the rest are rejected for growth (marked red).

The original algorithm for growing regions involves checking neighbouring voxels one by one. This means that the computation must be performed successively and thus cannot be parallelized [see Fig. 5 ▸(*a*)], leading to slow computation. Alternatively, and in order to enable parallel computing, we compute *C* and *D*_median_ for all the voxels within a bounding box and accept the ones fulfilling the growth criterion [see Fig. 5 ▸(*b*)]. Therefore, checking the availability of neighbouring voxels is not needed and the computation can be parallelized. For example, this can reduce the growth time for 10 000 voxels from about 400 to 2 s. Here, the length of the bounding box is determined by the sizes of the spots associated with the indexed seeding voxel for a specific geometry.

In summary, we have developed three approaches for the grain reconstruction: (i) CPU-*G*, using only CPU computing with *N*_*G*_ ranking and successive growth, which is slow but with the option to reconstruct and merge sub-volumes without using a GPU; (ii) GPU-*G*, using GPU computing with *N*_*G*_ ranking and independent growth; and (iii) GPU-*C*, using GPU computing with *C* ranking and independent growth. All the implementations were coded in MATLAB coupled with CUDA programming. The code has been published and is freely accessible (https://gricad-gitlab.univ-grenoble-alpes.fr/TomoX_SIMaP/GrainRecon).

## 3. Results

### 3.1. Comparison of grain reconstructions

We used different approaches to reconstructing the grains based on LabDCT projections in the Laue focusing and magnified geometries as shown in Fig. 3 ▸. The voxel size for the reconstruction was 2.5 µm, resulting in about 4.82 × 10^6^ voxels in the whole sample volume. Reconstruction parameters were set as *C*_trust_ = 0.85, max*D*_median_ = 10 pixels, max*D*_centre_ = 3 pixels and δ_drop-off_ = 0.02 for both geometries, whereas *C*_min_ = 0.55 for the Laue focusing geometry and *C*_min_ = 0.45 for the magnified geometry. These reconstruction parameters are typical values for a new grain reconstruction.

Table 2 ▸ summarizes the grain reconstruction results compared with the ground-truth input. All reconstructions render the same number of correctly indexed grains as the input, while other parameters, including average grain size, disorientation (Δ_OR_), differences in positions of grain centre of mass (Δ_COM,grain_) and individual grain size differences (Δ_D_), are all in good agreement with the input. Δ_OR_ and Δ_COM,grain_ are systematically smaller for reconstructions under the Laue focusing geometry than for the magnified geometry. This is primarily due to the use of a higher-resolution detector for the Laue focusing geometry, whereas it has little to do with the fact that more projections are used in the former geometry. To verify the latter statement, we performed an additional grain reconstruction with 181 projections in the magnified geometry (the number of spots per grain increases from ∼233 for 121 projections to ∼261 for 181 projections). The obtained grain reconstruction result has similar Δ_OR_ and Δ_COM,grain_ to those obtained with 121 projections, thus still showing inferior accuracy compared with the Laue focusing geometry.

As we pointed out earlier, the two simulated data sets of LabDCT projections mimic typical data sets obtained from two different tomography instruments. The aim of the comparison of grain maps is not to compare reconstruction performances under different geometries, but to demonstrate that all the current reconstruction methods are applicable to different geometries. Below, we select the reconstruction result using the method of GPU-*G* from projections in the magnified geometry to illustrate the comparison in more detail.

**Figure 6:**
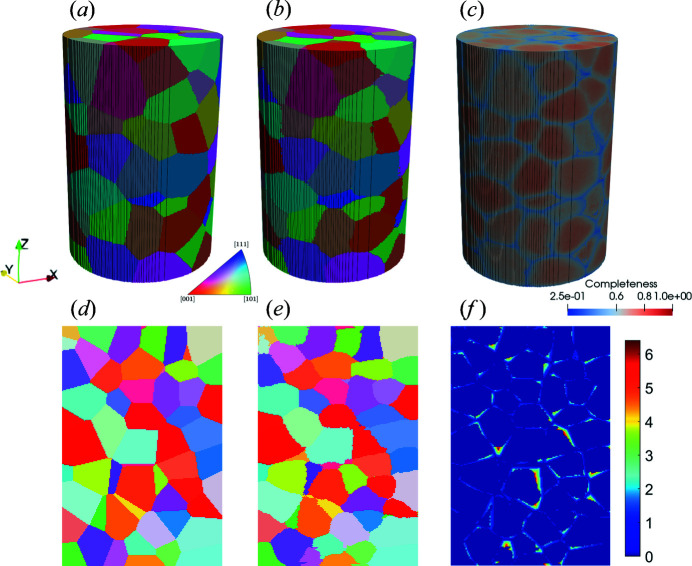
Comparison of grain maps. (*a*) A virtual input grain structure in a cylinder shape (diameter × height = 400 × 600 µm). (*b*) A reconstructed grain map using the GPU-*G* method from the LabDCT projections in the magnified geometry and (*c*) the corresponding completeness map. (*d*) A 2D *XZ* slice of the input. (*e*) A 2D *XZ* slice of the reconstructed grain maps. (*f*) A 2D *XZ* slice of the spatial deviation map, with a unit of pixels where 1 pixel = 2.5 µm. Note that the spatial deviations (ε_S_) for each pixel are computed in three dimensions using the method proposed by Fang, Hovad *et al.* (2021 ▸) but only visualized in two dimensions in panel (*f*).

Fig. 6 ▸ shows a comparison of grain maps between the input and the LabDCT reconstruction. In general, good agreements in grain shapes and orientations (indicated by the IPF-Z colours) can be seen in Figs. 6 ▸(*a*) and 6 ▸(*b*). Minor differences are observable at grain boundaries and, in particular, at grain junctions, where completeness values are relatively low [Fig. 6 ▸(*c*)]. Closer examination can be achieved with the 2D slices [Figs. 6 ▸(*d*) and 6 ▸(*e*)], and a quantitative map of spatial deviation is shown in Fig. 6 ▸(*f*). This last figure shows that most pixels have zero deviation, whereas the main deviating pixels lie close to grain boundaries, and the maximum deviation is found to be about 6.5 pixels for this slice.

**Figure 7:**
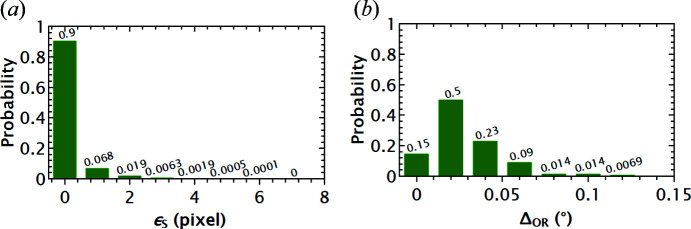
Histograms of (*a*) spatial deviation (ε_S_) for all sample voxels and (*b*) disorientation (Δ_OR_) for each grain with respect to the input ground truth.

A more quantitative comparison of both spatial and orientation accuracies can be seen in Fig. 7 ▸. Ninety per cent of the sample voxels are completely matched between the input and the reconstruction (ε_S_ = 0 pixels), while for 99% of voxels ε_S_ is no more than three pixels [Fig. 7 ▸(*a*)]. This confirms a rather good spatial accuracy. Fig. 7 ▸(*b*) shows that Δ_OR_ ≤ 0.07° for 95% of the reconstructed grains, whereas a few grains have disorientations up to 0.12°. By checking the forward simulated/projected spots (projections of the reconstructed grain volumes) overlaid onto the ‘experimental’ projections (ground truth), it was found that the spots for these grains have a higher frequency of intersecting with the overlapped spots compared with other grains. However, further analysis has not found any significant correlations between the more pronounced Δ_OR_ with either grain shape deviation or grain location (measured as the distance from the centre of mass to the rotation axis), although the latter was found to affect the reconstruction accuracy for an experimental LabDCT data set (Fang, Hovad *et al.*, 2021 ▸).

**Figure 8:**
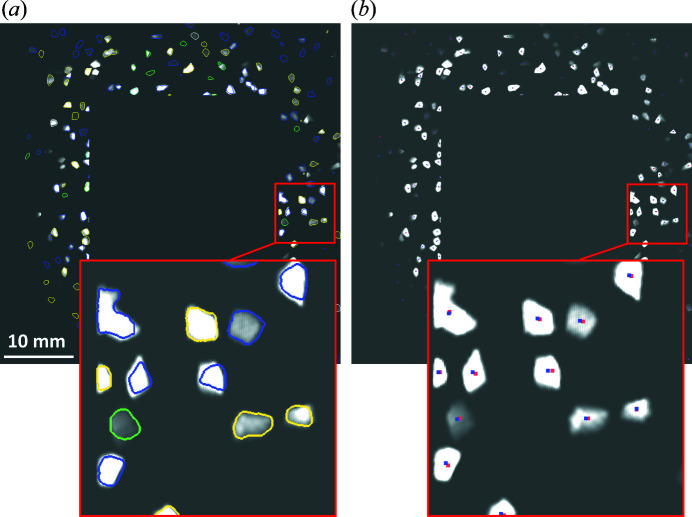
Checking forward spots on the LabDCT projection. (*a*) Outlines of the forward spots coloured according to {*hkl*} families and (*b*) positions of the centres of mass for forward (red points) and ‘experimental’ (blue points) spots overlaid onto the projection.

Another way of verifying the reconstructed grain map is to overlay forward projected spots on the ‘experimental’ projections, which is useful when there is no ground-truth grain map for comparison. Fig. 8 ▸(*a*) shows that the forward projected spot sizes, shapes and locations are in good agreement with the experimental spots. Visualization of the differences in spot centre positions allows a further assessment and quantification of the accuracy of the reconstructed grain map [Fig. 8 ▸(*b*)]. Minor differences can be seen more clearly in the zoom-in. The average difference in spot centre position was found to be about 2.3 ± 1.4 pixels based on statistics of 6940 spot pairs.

### 3.2. Computing time

**Figure 9:**
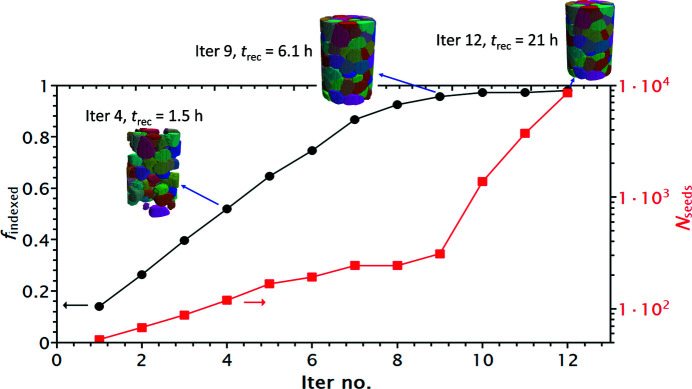
The reconstructed volume fraction (*f*_indexed_) and number of seeding voxels (*N*_seeds_) as a function of iteration number. The insets show 3D volumes obtained after the fourth, ninth and 12th iterations, respectively, and their reconstruction times.

To illustrate the grain mapping procedure, we plot the indexed volume fraction (*f*_indexed_) and number of seeds (*N*_seeds_) as a function of iteration number in Fig. 9 ▸. A greater iteration number corresponds to a finer grid for generating seeding voxels. The figure shows a linear increase in *f*_indexed_ for the first eight or nine iterations, after which *f*_indexed_ increases more slowly and reaches a maximum of about 0.980 while *N*_seeds_ increases exponentially. As illustrated in the figure, the first nine iterations were completed in 6.1 h with *f*_indexed_ = 0.956 and a total of 1484 seeding voxels. At this point, most of the grains have been correctly reconstructed, suggesting that the algorithm is efficient in reconstructing a near-complete volume. For a fast reconstruction one can stop the reconstruction early with a compromise on a few mis-indexings and small errors in the grain shapes. Nevertheless, it takes a much longer time to complete the remaining few percent of empty volumes with trials on many more seeding voxels. The reason is that in the later iterative computations most of the seeding voxels tend to be located close to grain boundaries, leading to more failures in the indexing, and thus more trials are needed.

It should also be noted that *f*_indexed_ has not reached 1 in the final iterations, suggesting that empty voxels remain in the sample. To solve this issue more quickly, rather than trying to index with many more seeding voxels (as shown by the stagnant increase in *f*_indexed_ in Fig. 9 ▸), we compute and compare the completeness for each empty voxel using orientations from indexed voxels within a certain distance (typically 20 pixels), from which the orientation giving the maximum completeness is assigned to the empty voxel (see Fig. 1 ▸). This significantly accelerates the computation speed, but also brings a risk of mis-indexing for the empty voxels. This is, however, considered to be tolerable when a large amount of computing time is saved, although in this specific case there were no mis-indexings. This completeness comparison approach has also been used for further checking of the voxels belonging to very small grains (*e.g.* <5 voxels) with too low a completeness.

## 4. Discussion

### 4.1. Characteristics of the current reconstruction methods

Comparisons of the reconstructed grain maps with the ground-truth data demonstrate that the algorithms can handle well LabDCT data with a large number of spots for different geometries. The reconstructed grains have good spatial and orientation accuracies. Although the reconstruction is currently limited to strain-free grains, the obtained grain map can be used as an input for resolving elastic lattice strains and intra-granular orientations, for which a fit to spot intensities will be required.

A well known limitation imposed by serious spot overlap applies to the current reconstruction methods, similar to many previously reported reconstruction methods for 3DXRD and its variants (*cf.* Lauridsen *et al.*, 2001 ▸; Ludwig *et al.*, 2008 ▸; Bernier *et al.*, 2011 ▸; Schmidt, 2014 ▸; Johnson *et al.*, 2008 ▸). As illustrated in the dis­orientation comparison, more spot overlap harms the accuracy of the indexed orientation, although moderate spot overlap is tolerable (in this work for the Laue focusing geometry, 13% of spots in the diffraction images contain more than one intensity peak, while the percentage is 18% for the magnified geometry). For a successful indexing with an error less than 0.1° the majority of the spots should not be connected with others. Spot segmentation is also critical to the reconstruction quality, even for the simulated projections with no background noise presented here, because point spread of spot intensities gives uncertainty for thresholding. Usually a Laplacian of Gaussian based segmentation works fine and, recently, a deep learning based spot segmentation has been demonstrated to be accurate, as well as less dependent on tuning of the segmentation parameters (Hovad *et al.*, 2021 ▸; Fang, Juul Jensen & Zhang, 2021 ▸).

### 4.2. Differences between the algorithms

The main features distinguishing the different reconstruction algorithms are (i) the way of ranking the orientation candidates for indexing and (ii) whether it is run with a GPU or only with a CPU. Each method has advantages and dis­advantages. As summarized in Table 3 ▸, the CPU-*G* method is slow but gives accurate reconstruction results and does not require a GPU, so is suitable for use with limited computing resources. We have implemented an acceleration for this method by cropping the full volume into sub-volumes for the reconstructions. Both the GPU-*G* and GPU-*C* methods are fast and accurate but they also have some disadvantages. The GPU-*G* method is more susceptible to spot overlap because it uses the spot centre of mass for back calculation, whereas it is less sensitive to geometric error because it compares the angles of the diffraction vectors. Conversely, the GPU-*C* method is more sensitive to geometric error, whereas it is less susceptible to spot overlap. The different features of the methods make them complementary to each other. For example, one may take advantage of the GPU-*G* method for grain reconstruction for a roughly known geometry, while using the GPU-*C* method for a data set with significant spot overlap.

All the proposed methods suffer from a common issue – the successful indexing rate decreases at later stages because seeding voxels are mainly found close to grain boundaries. To improve the indexing for ranking by *N*_*G*_, one option is to discard the spots associated with already-reconstructed grains. Therefore, wrong matches to these discarded spots can be avoided and the ranking of potential orientation candidates can be promoted. For ranking by *C*, the number of selected potential candidates can be increased to improve the successful indexing rate, or a finer global orientation sampling can be performed.

### 4.3. Influence of reconstruction parameters

The reconstruction parameters, particularly *C*_min_, have a significant influence on the final reconstruction. Too high a value of *C*_min_ may lead to too many false negatives (not-indexed voxels), whilst a *C*_min_ that is too low may cause too many false positives (wrongly indexed voxels). In this work, the value of *C*_min_ was set as a ‘standard’ for the magnified geometry (*C*_min_ = 0.45). It was set a bit higher for the Laue focusing geometry, due to the much higher average spot intensities, resulting in fewer spots being removed by intensity thresholding during the production of the diffraction projections. The parameter max*D*_median_ also affects the indexing when *C*_min_ is set below 0.5. An increased value for max*D*_median_ allows more successful indexing but also increases the risk of having more false-positively indexed voxels. Normally, max*D*_median_ should be set below 20 pixels and coordinated with the setting for *C*_min_.

The parameter *D*_median_ is found to be beneficial for the reconstruction accuracy for voxels with completeness values below 0.5, as shown in Section 2.3. Generally, low-completeness voxels correspond to relatively small grains and thus are associated with relatively weak diffraction spots, imposing a greater segmentation error compared with bright spots. However, this segmentation error can be compensated using *D*_median_ in combination with the completeness to compare the region growth.

Other parameters such as max*D*_centre_ and δ_drop-off_ also affect the region growth. max*D*_centre_ relates to the accuracy of the grain centre position. Usually, this parameter is set as three pixels, considering the balance of accuracy and computation time – a smaller value requires more iterations of the indexing and growth. The setting for δ_drop-off_ also has to take into account balancing the accuracy and the computation time – a value that is too high promotes too much over-growth, while a value that is too low makes the computation too slow. A value of 0.02 seems to be a reasonable setting in most cases.

The setting for the number of {*hkl*} families depends on the crystal structure. Three families are usually sufficient for a body-centred cubic crystal, while four are preferred for a face-centred cubic crystal. For other crystal structures with lower symmetry, at least four {*hkl*} families are required.

### 4.4. Other potential reconstruction approaches

Current reconstruction methods are based on a grain-by-grain approach, *i.e.* indexing one orientation followed by expanding this orientation to other voxels. Our methods can be adapted further to other potential approaches that separate the indexing and the growth completely, *i.e.* which index all possible orientations first using the method presented here, and then reconstruct the grain shape for each indexed orientation. The latter can be realized either by tomographic back projection or by comparing completeness for neighbouring orientations.

The tomographic back projection approach has been successfully implemented in synchrotron DCT with the simultaneous iterative reconstruction technique using the 3D model of the *ASTRA* toolbox (Ludwig *et al.*, 2009 ▸; Reischig *et al.*, 2013 ▸; van Aarle, Palenstijn *et al.*, 2015 ▸). It was also used in the very first reconstruction approach with LabDCT (King *et al.*, 2013 ▸), which required an affine transformation of spot shapes accounting for astigmatism (the magnifications for the projected spots are different in directions parallel and perpendicular to the diffraction vector). The implementation of an accurate polychromatic cone-beam projection model was proposed by van Aarle, Ludwig *et al.* (2015 ▸). Other than that, there will be an issue with empty voxels and gaps between grains after the back projection. Whilst a dilation approach has been used in synchrotron DCT, a more physics-based approach would be to compare the completeness (and *D*_median_ if the computational cost does not increase too much) using the orientation candidates nearby, as we also use in our current methods for completing the final volume (see Section 3.2).

The other approach is based on a brute force indexing for a uniform and fine sample grid. After the indexing, non-indexed voxels will be assigned by one of the neighbouring orientations that gives a maximum completeness. We implemented this approach by setting a constant box size of 20 pixels for searching the candidates. While a similar grain reconstruction can be obtained, this approach requires much more computation time (because of a finer sample grid) and often causes noisy single voxels (with orientation different from neighbouring voxels), being slower and less robust. However, optimization for the generation of seeding voxels and searching the range of orientation candidates may improve this approach.

In parallel with other grain mapping algorithms introduced in the review papers (Poulsen, 2012 ▸; Reischig & Ludwig, 2020 ▸) and the references therein, our methods are open source and are intended to foster the development of the laboratory-based grain mapping technique, and other related synchrotron white or pink beam based diffraction imaging techniques, complementary to the current well established techniques using a monochromatic synchrotron beam.

## 5. Conclusions

Multiple grain reconstruction algorithms based on forward and back calculations have been presented in detail. Differing in their indexing strategy and computational implementation, three reconstruction methods have been developed, among which an efficient computation is achieved with GPU parallel computing.

These grain reconstruction methods have been demonstrated on a synthetic data set containing 144 grains and moderate spot overlap for both Laue focusing and magnified geometries. Comparisons of grain maps show that, on average, the reconstructed orientation accuracy is 0.03°, the error in the grain centre-of-mass position is within two pixels and the relative grain size difference is 4%.

These methods are not limited to a specific instrument and can be applied to various types of LabDCT data sets acquired on different instruments. Possibilities for extending the current algorithms to other reconstruction methods have also been presented.

## Figures and Tables

**Figure 10:**
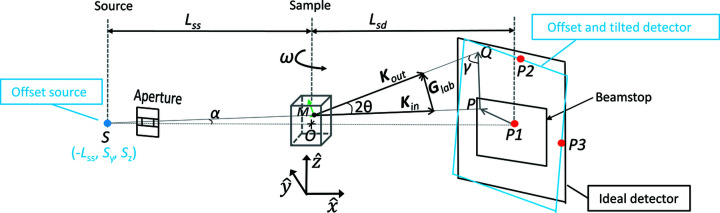
A schematic overview of the LabDCT technique with the setup geometry defined in a laboratory right-handed coordinate system ( 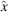 , 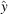 , 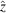 ). The incoming beam, emitted from a point source (*S*), travels through an aperture and illuminates the sample, and the detector records the transmitted signals. For a diffraction event occurring at the sample position *M*, the incoming wavevector **K**_in_ and scattered wavevector **K**_out_ together determine the scattering vector **G**_lab_. Position *Q* indicates the intersection point of the diffracted beam with an ideal detector and 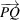 stands for the projection of **G**_lab_ on the detector. *P*_1_, *P*_2_ and *P*_3_ indicate the centre, the centre of the top edge and the centre of right edge of the detector, respectively. Note that the dimensions are not to scale.

**Table 1:** Geometric information for simulating LabDCT projections under Laue focusing and magnified geometries See Appendix *A* [App appa] for the notation used.

Geometry	*L*_ss_ (mm)	*L*_sd_ (mm)	det*y*0 (mm)	det*z*0 (mm)	φ_*x*_ (°)	φ_*y*_ (°)	φ_*z*_ (°)	Detector width × height, pitch size (µm pixel^−1^)
Laue focusing	11	11	0	0	0	0	0	2032 × 2032, 3.36
Magnified	6.14	52.89	−0.24	1.59	0.01	0.64	0.35	2040 × 2040, 24

**Table 2:** Summary of grain reconstructions compared with the ground truth 〈*D*〉 is the average grain diameter, *N*_grain_ the number of grains, *N*_total_ the total number of indexed grains, *N*_correct_ the number of correctly indexed grains, Δ_OR_ the average disorientation for grain pairs, Δ_COM,grain_ the average distance between grain centroids, Δ_D_ the relative grain size difference and *t*_rec_ the reconstruction time. Values are expressed by a mean value with or without a standard deviation. The CPU-*G* result was obtained using a computing node with 28 CPU cores.

			*N*_grain_					
Data set or geometry	Method	〈*D*〉 (µm)	*N*_total_	*N*_correct_	Δ_OR_ (°)	Δ_COM,grain_ (pixel)	Δ_D_	*t*_rec_ (h)
Ground truth	–	98.6 ± 11.1	144	–	–	–	–	–
Laue focusing	CPU-*G*	98.2 ± 13.1	144	144	0.019	1.7 ± 0.6	0.035	186
	GPU-*G*	98.1 ± 13.4	144	144	0.018	1.7 ± 0.6	0.043	22
	GPU-*C*	98.5 ± 13.8	144	144	0.017	1.9 ± 0.8	0.044	22
Magnified	GPU-*G*	98.4 ± 12.3	144	144	0.038	2.2 ± 0.9	0.041	21
	GPU-*C*	98.5 ± 12.2	144	144	0.034	2.1 ± 0.8	0.031	21

**Table 3:** Summary of advantages and disadvantages for different grain reconstruction methods ‘OR’ stands for orientation.

Method	Advantages	Main disadvantages	Comments
CPU-*G*	(i) No need for GPU	Slow	Full volume can be cropped into sub-volumes for reconstruction
GPU-*G*	(i) Fast	More susceptible to spot overlapping	Discard spots associated with already-reconstructed grains during 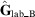 calculation
	(ii) Less sensitive to geometric error		
	(iii) Coarser OR discretization		
GPU-*C*	(i) Fast	(i) More sensitive to geometric error	Increased number of selected potential candidates, *N*_candidates_
	(ii) Less susceptible to spot overlapping	(ii) Finer OR discretization	

## Appendix A

### A1. Setup geometry

Fig. 10 ▸ shows an overview of the LabDCT geometry in a conventional system. All coordinates are defined in a right-handed laboratory frame, for which the origin is located on the rotation axis and sits inside the sample. The  axis is along the beam, the  axis is transverse to the beam in the horizontal plane and the  axis is along the vertical rotation axis. Ideally, the X-ray source aligns with the origin and detector centre, and the detector is perpendicular to the X-ray beam. In practice, however, both the source and the detector may be offset horizontally and vertically. In addition, the detector may not be perpendicular to the beam, *i.e.* it may be tilted with respect to the beam.

To generalize the geometry, let us define the X-ray source position as (−*L*_ss_, *S*_*y*_, *S*_*z*_) and the detector centre position as (*L*_sd_, det*y*0, det*z*0). For a tilted detector with respect to the incoming beam along the direction (1, 0, 0), we define φ_*x*_, φ_*y*_ and φ_*z*_ as the tilt angles about the *x*, *y* and *z* axes, respectively (being positive in a counterclockwise direction). The corresponding rotation matrices *R*_*x*_, *R*_*y*_ and *R*_*z*_ are

The total rotation can thus be expressed as **R**_det_ = *R*_*z*_ *R*_*y*_ *R*_*x*_ as a 3 × 3 matrix. For an ideal geometry, *S*_*y*_ = *S*_*z*_ = 0, det*y*0 = det*z*0 = 0 and φ_*x*_ = φ_*y*_ = φ_*z*_ = 0°. For a tilted detector, the tilt angles can either be calculated from the coordinates of three orthogonal positions, *e.g.* as indicated by *P*_1_, *P*_2_ and *P*_3_ in Fig. 10 ▸, if such information is encoded by the measured data, or be preset to zeros and further refined by subsequent fitting on the geometry based on the initial grain mapping result.

As shown in Fig. 10 ▸, the scattering vector **G**_lab_ in the laboratory coordinate system for an (*hkl*) plane of a grain fulfilling the Bragg diffraction condition can be determined as

where Ω is a matrix transforming a rotated system to the laboratory system, *T* is a matrix transforming a sample system to the rotated system, *g* ^−1^ is a matrix transforming a Cartesian crystal system to the sample system, *i.e.* crystal orientation, *B* is a matrix transforming reciprocal space to the Cartesian crystal system and  . The formulation of these transformation matrices is presented by Poulsen (2004 ▸).

The incoming wavevector **K**_in_ of the diffraction event occurring at a sample position  can be expressed as

where λ_*hkl*_ is the photon wavelength that fulfils Bragg’s law. The scattered wavevector **K**_out_ can thus be expressed as

The unit vector of **K**_out_ can be written as

Given the diffraction position *M* and **G**_lab_, to work out the position where **K**_out_ hits the detector is usually referred to as forward calculation, whereas computation of **G**_lab_ given *M* and the intersection position of the diffracted beam on the detector is called back calculation. Several forward projection models for LabDCT have been reported (van Aarle, Ludwig *et al.*, 2015 ▸; Niverty *et al.*, 2019 ▸; Fang *et al.*, 2020 ▸), while a method for dealing with a non-ideal setup geometry has yet to be presented in detail. In the following, a generalized framework of forward and back calculations considering offsets and tilts (which are often present in practice) is presented. This framework is partly inspired by a geometric treatment for 3DXRD (Wright, 2005 ▸). Since the proposed grain reconstruction works on binarized spot images, the calculation of signal intensity is not required and will not be presented here; it has previously been reported in detail by Fang *et al.* (2020 ▸).

### A2. Forward calculation

We first derive the expected intersection position of **K**_out_ on the detector without any offset and tilt (ideal case). Then, we take into account the detector offset and tilt and subsequently derive the actual intersecting position.

Given the sample position  and **G**_lab_, the expected intersection position *Q* (*L*_sd_, *y*_det_ideal_, *z*_det_ideal_) on an ideal detector can be determined using

where *y*_*p*_ and z_*p*_ are given by

and the length of the diffraction displacement *L*_diff_ (  in Fig. 10 ▸) can be calculated using

Here,

is the angle between  and  , and γ is the angle between  and **K**_out_ (see Fig. 10 ▸). The angle γ can be calculated as

where (*L*_ss_ + *L*_sd_, *y*_*p*_, *z*_*p*_) is the vector  and [0, **G**_lab_(2), **G**_lab_(3)] is a vector parallel to  .

Given the derived position for *Q* (*L*_sd_, *y*_det_ideal_, *z*_det_ideal_), we can now rewrite the unit vector of **K**_out_ as

Considering the offset and tilt of the detector, the intersection positions (det*y*_F_, det*z*_F_) on the offset and tilt detector fulfil

From this equation, *t* can be solved as

where *R*_*ij*_ (*i*, *j* = 1, 2 or 3) denotes an element of **R**_det_. Thus, the intersection positions (det*y*_F_, det*z*_F_) can be derived as

Here, det*y*_F_ and det*z*_F_ are expressed as coordinate values in the detector frame with its origin sitting at the centre of the detector. Now it becomes straightforward to convert (det*y*_F_, det*z*_F_) to pixel coordinates by taking account of the pixel pitch and the width and height of the detector.

### A3. Back calculation

Given the sample position  and a diffraction signal position (det*y*_B_, det*z*_B_) on the tilted detector specified in the detector frame, we can back calculate **G**_lab_. First, we convert the detector centre (*L*_sd_, det*y*0, det*z*0) specified in the detector frame to the laboratory frame (  ),

where **d** is a 3 × 1 matrix and can be calculated as **d** =  . Then, the unit vector of **K**_out_ from the back calculation can be derived as

Thus, the unit vector of **G**_lab_ can be computed as

where

For a specific {*hkl*} with a lattice spacing of *d_hkl_* leading to the diffraction, the magnitude of **G**_lab_ equals 2π/*d_hkl_*. For the sample position *M*, one can now compare the angles of **G**_lab_ between the back [expressed in equation (20)] and the forward calculations [expressed in equation (4)].
